# Microscopic hematuria and pelvic ultrasonography could rule out flexible cystoscopy during surveillance for T1-low grade non-muscle invasive bladder cancer

**DOI:** 10.1080/2090598X.2023.2202930

**Published:** 2023-04-17

**Authors:** Mohamed Awad, Ahmed M. Harraz, Hashim Farg, Hady S. Gabr, Doaa E. Sharaf, Mohamed Abou-El-Ghar, Ahmed S. El-Hefnawy, Yasser Osman

**Affiliations:** Urology and nephrology center, Mansoura University, Mansoura, Egypt

**Keywords:** Non-muscle invasive bladder cancer, hematuria, surveillance, ultrasonography, cystoscopy

## Abstract

**Purpose:**

Cystoscopy (rigid/flexible [FC]) is the standard surveillance tool for non-muscle invasive bladder cancer (NMIBC). Nevertheless, it has its drawbacks. The objective of this study is to evaluate the performance of microscopic hematuria (MH), abdominal ultrasonography (US), and urine cytology (UC) as potential substitutes for FC in patients with T1-low-grade (T1-LG) NMIBC.

**Methods:**

Over a 12-month period, patients attending our tertiary referral center for T1-LG NMIBC follow-up underwent urine analysis for MH and UC, and then US and FC were performed as outpatient surveillance procedures. Those with positive findings underwent inpatient rigid cystoscopy under anesthesia and biopsy. The negative predictive values (NPV) and sensitivity of different combinations of MH, UC, US, and FC were compared with the standard histopathology.

**Results:**

In 218 evaluated patients, FC had the highest NPV (97.9%). However, this figure showed no statistically significant difference if compared with the combination of negative MH and US (93.8%) (difference = 0.04, *p* = 0.1) or the combination of MH, US, and UC (94.9%) (difference = 0.03, *p* = 0.2). The reported sensitivity results were similarly comparable between FC (94.2%) and the aforementioned combinations (90.4% and 92.3%; differences: 0.038 and 0.019; *p* = 0.4 and 0.7, respectively).

**Conclusions:**

During the surveillance of NMIBC for patients diagnosed with T1-LG disease, the combination of MH/US has comparable sensitivity and NPV with FC. This non-invasive combination could be considered the first station that might preclude the need for FC in a considerable percentage of this group of patients.

## Introduction

Non-muscle invasive bladder cancer (NMIBC) is present in approximately three-fourths of patients diagnosed with bladder cancer, and it is estimated that 20–40% will be at high risk of disease recurrence and progression [1]. The risk groups can be determined based on the European Organization for Research and Treatment of Cancer (EORTC) scoring system or according to the conjoint guidelines of the American Urological Association (AUA), Society of Urologic Oncology (SUO), and the European Association of Urology [2,3].

Surveillance after the initial resection is an integral part of the management protocol and should reflect the individual patient’s degree of risk. In most guidelines, it is agreed that follow-up should be done every 3–6 months in the first 2 years and then every 6 months to 1 year for the next couple of years and then yearly thereafter [3,4], and more importantly, the clock should be reset after each recurrence. Although the mainstay procedure for surveillance is outpatient flexible cystoscopy (FC), it is not devoid of its morbidity. First, patient anxiety, pain, and discomfort have been reported with many methods that have been described to mitigate such feelings [5,6]. Urinary tract infection is another possible morbidity in this target group during invasive cystoscopic surveillance [7]. Furthermore, cost-effectiveness is a concern, especially for older adults [8,9]. Finally, the necessity of non-invasive surveillance was raised during this COVID-19 pandemic to avoid a delay that might affect the oncological outcome [10].

In addition to cystoscopy, urine cytology (UC) has a pivotal role notably in high-grade disease and might improve surveillance accuracy [11,12]. Besides, a handful of urinary biomarkers were investigated to optimize surveillance protocols for NMIBC [13]. Nevertheless, its use is limited by its modest accuracy as well as the high cost, while the clinical effectiveness might be ruined by factors such as BCG therapy [14].

To combat the FC limitations, and for the sake of improving the patient experience during follow-up, we aimed to prospectively compare the performance of a combination of three daily used, non-invasive measures, namely microscopic hematuria (MH), abdominal ultrasonography (US), and UC, and as possible substitutes for FC in patients undergoing surveillance for T1-low-grade (T1-LG) NMIBC.

## Methods

### Study design

A retrospective analysis for prospectively collected data was conducted for patients undergoing surveillance after TURBT for T1-LG NMIBC in a tertiary referral center over a period of 12 months. The protocol of the study was approved by the institutional review board before patient enrollment, and written informed consent was taken (Mansoura Faculty of Medicine IRB: MS/15.08.68). The authors confirm the availability of, and access to, all original data reported in this study. All patients who underwent TURBT with initial pathology of T1-LG disease and were scheduled for surveillance were included. Grading was evaluated according to the WHO 2004 grading system. Patients with biopsy-proven high-grade or muscle-invasive disease, carcinoma in situ, gross hematuria, persistently positive urine culture, or had contracted bladder were excluded. All of our patients received immediate intravesical instillation of Epirubicin. Maintenance intravesical instillation was continued in due patients according to the EUA guidelines.

### Measurements

Demographics included age, gender, associated comorbidities, body mass index (BMI), and the time since the last TURBT. Urine clean catch samples were withdrawn before and after FC to assess the incidence of UTI. Patients with positive urine culture received the appropriate antibiotics, and the culture was rendered negative before the procedure. For the assessment of patients’ experience, a visual analog score (VAS) was obtained during FC, and a question was asked (satisfied/unsatisfied). Additionally, IPSS data before and 1 week after FC were collected.

### Intervention

Initially, patients underwent urine analysis for MH followed by referral to a dedicated uro-radiologist for transabdominal pelvic US, and then an appointment was scheduled to undergo outpatient FC during which UC was obtained. Patients with negative findings of all the tests were discharged and considered negative, while inpatient cystoscopy and biopsy were performed if any of the test results were positive.

Urine samples were collected and examined for hematuria by microscopic examination of the urine sediment. For cytological evaluation, urine samples were collected by bladder washing with saline before FC and were sent for cytology. Urine was centrifuged, cyto-spinned, and stained with Papanicolaou stain and examined by an academic pathologist. The US was performed by a dedicated radiologist who was blinded to the findings of the other tests, and changes in the lining mucosa were described. Then, patients were subjected to FC by a dedicated experienced urologist. In the indicated patients, rigid cystoscopy under anesthesia was then carried out, and biopsy from any suspicious area and multiple random biopsies from the rest of the bladder mucosa were taken and sent for histopathology.

### Outcome

The research question was how much negative MH, US, and UC were accurate in excluding the presence of bladder cancer and consequently might ameliorate the need for FC. Hence, we compared primarily the negative predictive value (NPV) of MH, UC, and US and their relative combinations in comparison to FC for the detection of recurrent bladder cancer. Positive test results were considered for MH if ≥3 RBCs/HPF was present as suggested by the AUA guidelines [15], and for UC if high-grade malignant urothelial cells were detected, while for US if a focal mural thickening or intra-luminal growth was noted. Regarding FC, the findings were considered positive if any abnormal changes in the mucosa, e.g. red, velvety, polypoidal gross, or solid mass, were detected. The result of in-patient biopsy was considered positive when evidence of malignancy is detected in the specimens. Patients with negative MH, UC, US, and FC were considered devoid of malignancy. The secondary outcome of the study is to assess patient satisfaction during FC and newly developed UTI following the FC.

### Statistical analysis

All data were prospectively collected and maintained in an electronic database. The sensitivity of a test is defined as the percent of patients with the disease for whom the test is positive (true positive/total number of patients with the disease × 100). A comparison of sensitivity between the two measures was performed using the McNemar test. The negative predictive value (NPV) is defined as the percent of individuals in whom the test is negative, and the disease is not present [true negative/test negative (true negative + false negative) × 100]. Statistical differences of NPV were calculated with the generalized score statistic test. Specificity and PPV were not the primary targets of the study. Wilcoxon signed-rank test was used for paired comparison of non-parametric variables. Statistical analysis was performed using IBM v.25 statistical software and R programming language v. 3.6.3 (http://www.r-project.org) with the package *DTComPair*.

## Results

A total of 218 patients, 202 males representing 92.6%, were included in the study. The median (IQR) IPSS score before FC was 11 (6, 20) and has increased to 14 (7, 20) 1 week after the FC (*p* = 0.01). Thirty-six (17%) patients showed positive urine culture following FC. The median (IQR) VAS was 7 (4–8), and 87% of the patients were not satisfied regarding the FC experience. Table 1 summarizes the demographics and the detailed results of MH, UC, US, and FC.

**Table 1.** Demographic and diagnostic test results in patients undergoing surveillance for T1-low-grade non-muscle invasive bladder cancer.

Variable	Level	Total (*n* = 218)
Age, years	Mean (SD)	59.9 (10.2)
BMI	Mean (SD)	29.8 (7.9)
Diabetes mellitus	No	172 (78.9)
	Yes	46 (21.1)
Hypertension	No	162 (74.3)
	Yes	56 (25.7)
Time since TURBT, months	Median (IQR)	19 (6.0, 46.8)
Time since TURBT, years	<1 yr	89 (40.8)
	1–2	39 (17.9)
	2–3	25 (11.5)
	3–4	15 (6.9)
Details of the diagnostic tests		
Microscopic hematuria	No	132 (60.6)
	Yes	86 (39.4)
Transabdominal US	Negative	106 (48.6)
	Diffuse thickening	48 (22.0)
	Focal thickening	19 (8.7)
	Nodule/lesion	45 (20.6)
Cytology	Hyperplastic smear	38 (17.4)
	Inflammatory smear	148 (67.9)
	Malignant smear	17 (7.8)
	Suppurative	5 (2.3)
	Atypical	4 (1.8)
	Acellular	6 (2.8)
Outpatient flexible cystoscopy	Negative	147 (67.4)
	Suspicious area	3 (1.4)
	Recurrent papillary lesion	68 (31.2)

TURBT: Transurethral resection of bladder tumor; BMI: Body mass index; US: Ultrasound

*Of the primary tumor

FC was falsely negative in three patients who had tiny single papillary growths (<1 cm). Two lesions (pTa) were missed being in the trigone close to the bladder neck in elderly patients with a bulging median lobe. Another lesion (pT1) was located in the anterior wall close to the bladder neck. These lesions were detected by ultrasound examination as focal intraluminal growth and two were tested positive for MH. On the other hand, the combination MH/US was falsely negative in five patients; all were small (<1 cm) papillary lesions (3 pTa and 2 pT1) and were detected by FC. The addition of UC to the MH/US combination excluded one of these patients.

Among MH, UC, US, and FC, the latter had the highest NPV (97.9%). However, this figure showed no statistically significant difference if compared with the combination of MH and US (93.8%) (difference = 0.04, *p* = 0.1) or the combination of MH, US, and UC (94.9%) (difference = 0.03, *p* = 0.2). The reported sensitivity results were similarly comparable between FC (94.2%) and the previous combinations (90.4% and 92.3%; differences: 0.038 and 0.019; *p* = 0.4 and 0.7, respectively). Table 2 depicts the performance of the different diagnostic tests in comparison to the final histopathology.

**Table 2.** The performance of the diagnostic test results and the final histopathology in patients undergoing surveillance for T1-low-grade non-muscle invasive bladder cancer.

Variable	Level	Benign (*n* = 166)	Urothelial carcinoma (*n* = 52)
MH	Negative	112 [85%]	20
	Positive	54	32 (62%)
UC	Negative	165 [82%]	36
	Positive	1	16 (31%)
US	Negative	100 [94%]	6
	Positive	66	46 (89%)
Negative MH/US	Negative	76 [94%]^#^	5
	Positive	90	47 (90%) *
Negative MH/US/UC	Negative	75 [95%]^##^	4
	Positive	91	48 (92%) **
FC	Negative	143 [98%]	3
	Positive	23	49 (94%)

MH: Microscopic hematuria (>3 RBCs/HPF); US: transabdominal pelvic US; UC: Urine cytology; FC: Flexible cystoscopy.

Values in () represent the sensitivity; Values in [] represents the negative predictive values (NPV).

*difference with FC sensitivity is 0.038, *p* = 0.4; **difference with FC sensitivity is 0.019, *p* = 0.7.

^#^difference with FC NPV is 0.04, *p* = 0.1; ^##^difference with FC NPV is 0.03, *p* = 0.2.

## Discussion

Generally, the surveillance test depends upon the target population to be studied. If a marker is planned to be used for bladder cancer screening in the general population, the primary target is to have a high positive predictive value to reduce anxiety and further expensive investigations in false-positive cases. Conversely, if a marker is to be used for monitoring a high-risk population, the marker must have a high negative predictive value to detect each case of the targeted pathology [16]. Our study confirmed the well-established fact that FC is an excellent diagnostic test for monitoring bladder tumor recurrence in high-risk patients with an excellent negative predictive value. Nevertheless, many pitfalls of this invasive long-lasting diagnostic procedure were described.

Despite all technological advances, Koo et al. recently reported that two-thirds of patients under surveillance for NMIBC experienced a variable degree of procedural discomfort or worry; hence, attempts to alleviate the process-related stress are ongoing [17]. Therefore, a plenty of tools have been described to mitigate pain and anxiety, including listening to music and increasing irrigation pressure during the procedure [5,6]. Our data showed that most of our patients were unsatisfied with the procedure with a significant increase of the urinary symptoms following FC up to 1 week after the procedure, mostly due to the irritation induced by the FC in an elderly group of male patients with prostatic enlargement. Furthermore, urethral pain and anxiety might be associated with incomplete bladder examination and patient non-compliance, particularly in the elderly and those with benign prostatic obstruction. In this experience, three patients with bladder malignancy were missed during outpatient FC. Similarly, it has been reported that FC could miss up to 10% of lesions; therefore, photodynamic fluorescence techniques were implemented to improve the diagnostic ability in continuous strive for improving the accuracy of FC [14].

We elected to include infection-free patients and those with persistent UTIs were excluded from our protocol. The aim is to accurately determine the incidence of *de novo* infection-related to FC. Although 18% of our patients showed evidence of newly acquired UTIs following FC, the clinical significance of this bacterial infection was limited, and none of them was hospitalized for urosepsis. Herr reported that only 2% developed febrile UTIs following this invasive outpatient procedure [7]. We believe that this incidence should not be underestimated and might constitute a significant health burden in this elderly population that might need a lifelong follow-up. However, a published meta-analysis did not recommend routine antibiotic prophylaxis in patients who undergo cystoscopy with sterile urine in the ambulatory setting [18]; nevertheless, this necessitated that urine culture is performed for every patient which might create an extra burden.

Currently, the calculation of the cost-effectiveness of any diagnostic and interventional procedure constitutes a pivotal role in sound medical practice. In fact, surveillance cost explains why NMIBC is considered one of the most expensive cancers to manage [19]. Mossanen et al. calculated the annual cost of surveillance for low-risk NMIBC to be $3297 and $1649 for the first and subsequent years, while the annual costs of surveillance were $6594 for the first 2 years and $3297 for subsequent years for intermediate- and high-risk diseases [20]. The costs of the combined ultrasound and urine analysis are considered extremely modest compared to FC. On the other hand, UC is not considered a cheap diagnostic tool for this pathology surveillance [21]. Interestingly, our data provided evidence that the exclusion of the UC from the suggested combination algorithm did not compromise its diagnostic ability.

We have included patients with T1-LG disease and excluded high-grade diseases to attempt to improve the sensitivity and NPV of the diagnostic combination tests. According to the EAU guidelines, most of the tumors infiltrating the lamina propria would require lifelong surveillance [22]. Nevertheless, a very high-risk group is added which included the combination of high-grade with TI and the presence of CIS. Hence, we opted to exclude this high-risk disease having had a higher risk of recurrence as well as progression.

Urine analysis for MH was included for being readily available, non-invasive, and as MH is closely pertinent to urothelial cancers [23]. According to the AUA guidelines, adult patients >35 years presenting with asymptomatic MH should undergo a cystoscopic evaluation of the bladder [15]. In a recent observational study of 2,166 patients, bladder cancer was detected by US in 11% and 2.7% presented with macroscopic and microscopic hematuria, respectively [24]. During the active surveillance of 55 low-risk NMIBC patients, hematuria was reported in 16.4% during the follow-up period, and it was the reason for follow-up discontinuation and scheduling for TURBT [25]. In the current report, the NPV of MH alone was relatively low (84.8%) and could not be utilized as a solo surveillance test.

More than three decades ago, the transabdominal US was suggested as a non-invasive, cost-effective diagnostic for surveillance of NMIBC [26,27]. Nevertheless, this approach has never gained popularity. The current technological advances and the development of sensitive US probes have skyrocketed the accuracy of intraluminal visualization and improved bladder cancer detection including options such as 3D-US and color Doppler modes [28]. In the current era, two separate groups of investigators prospectively evaluated the role of US in NMIBC surveillance. Interestingly, both reported a nearly matched NPV (85% and 86%, respectively) [29,30]. In our experience, a higher value (94.3%) was recorded probably because of the inclusion of a dedicated radiologist in this investigation.

Although the addition of MH to US has reduced the number of falsely negative lesions by only one, it is important to highlight the merits of adding MH to US. First, urine analysis is routinely performed in everyday practice, so it would not add a significant extra-cost for the diagnosis. In addition, MH has improved the results, and consequently, its importance might emerge in large cohorts of patients.

We introduce a non-invasive, readily available, and cheap combination for the surveillance of NMIBC that would be considered the first station. If both modalities yielded negative results, in this case scenario, FC could be precluded. On the other hand, if any had positive results, FC should be recommended. In this study, this combination could exempt approximately one-third of patients from doing FC without potentially compromising cancer detection. Further studies with a combination of urinary biomarkers might heighten the accuracy of the proposed combinations.

Nevertheless, the study is not devoid of its weakness. We did not account for approved urinary biomarkers, which have shown clinical significance in the surveillance of NMIBC [13]. We acknowledge that the incidence of low-grade T1 tumors in non-muscle invasive bladder cancer is not high but not rare and represent about 15% to 40% that continue to vary according to the institution [31]. Another drawback of our study is the lack of evaluation of lymphovascular invasion and variant histology. In addition, we acknowledge that adopting a new strategy that would eliminate FC from clinical practice is hard to accept by many clinicians; however, this report suggested that this could be safely performed in a highly selected group of patients.

## Conclusions

The combination of negative transabdominal US, the absence of MH in urine analysis, and optionally negative cytology could be safely considered as the first station for NMIBC surveillance in patients with T1-LG disease. This approach might mitigate the need for FC in this selected group of patients without compromising cancer detection. Further studies are needed to validate these findings.

## Limitations of the study

1. The use of T1 low-grade NMIBC only
2. Not using the new approved urinary biomarkers
3. The lack of evaluation of lymphovascular invasion and variant histology.
